# Prognostic factors for the occurrence of post-operative shoulder stiffness after arthroscopic rotator cuff repair: a systematic review

**DOI:** 10.1186/s12891-022-05030-4

**Published:** 2022-01-28

**Authors:** Thomas Stojanov, Linda Modler, Andreas M. Müller, Soheila Aghlmandi, Christian Appenzeller-Herzog, Rafael Loucas, Marios Loucas, Laurent Audigé

**Affiliations:** 1 Department of Orthopaedic Surgery and Traumatology, University Hospital of Basel, Basel, Switzerland; 2 Research and Development, Shoulder and Elbow Surgery, Schulthess Clinic, Zurich, Switzerland; 3 Basel Institute for Clinical Epidemiology and Biostatistics, University Hospital Basel and University of Basel, Basel, Switzerland; 4 University Medical Library Basel, University of Basel, Basel, Switzerland; 5 Department of Orthopedics, Balgrist University Hospital, University of Zurich, Zurich, Switzerland

**Keywords:** Risk factors, Prognostic factors, Arthroscopy, Rotator cuff tear, Shoulder stiffness

## Abstract

**Background:**

Post-operative shoulder stiffness (POSS) is one of the most frequent complications after arthroscopic rotator cuff repair (ARCR). Factors specifying clinical prediction models for the occurrence of POSS should rely on the literature and expert assessment. Our objective was to map prognostic factors for the occurrence of POSS in patients after an ARCR.

**Methods:**

Longitudinal studies of ARCR reporting prognostic factors for the occurrence of POSS with an endpoint of at least 6 months were included. We systematically searched Embase, Medline, and Scopus for articles published between January 1, 2014 and February 12, 2020 and screened cited and citing literature of eligible records and identified reviews. The risk of bias of included studies and the quality of evidence were assessed using the Quality in Prognosis Studies tool and an adapted Grading of Recommendations, Assessment, Development and Evaluations framework. A database was implemented to report the results of individual studies. The review was registered on PROSPERO (CRD42020199257).

**Results:**

Seven cohort studies including 23 257 patients were included after screening 5013 records. POSS prevalence ranged from 0.51 to 8.75% with an endpoint ranging from 6 to 24 months. Due to scarcity of data, no meta-analysis could be performed. Overall risk of bias and quality of evidence was deemed high and low or very low, respectively. Twenty-two potential prognostic factors were identified. Increased age and male sex emerged as protective factors against POSS. Additional factors were reported but do require further analyses to determine their prognostic value.

**Discussion:**

Available evidence pointed to male sex and increased age as probable protective factors against POSS after ARCR. To establish a reliable pre-specified set of factors for clinical prediction models, our review results require complementation with an expert's opinion.

**Supplementary Information:**

The online version contains supplementary material available at 10.1186/s12891-022-05030-4.

## Background

Patients expect the highest level of safety and effectiveness when they undergo elective orthopedic surgery. Satisfied pre-operative expectations as to safety and effectiveness of an orthopedic procedure are among the main determinants of patient satisfaction post-operatively [1].

Patient safety in surgery involves issues related to the quality of care, the occurrence of adverse events (AE), and their management. Published rates of AEs in orthopedics are variable [2, 3]. Following arthroscopic rotator cuff repair (ARCR), recurrence of rotator cuff defects, worsening or persisting pain or post-operative shoulder stiffness (POSS) are the most prevalent AEs. POSS, which affects 5 to 10% of patients [4], may remain mild, but can also cause severe functional disability in everyday activities, requiring prolonged rehabilitation and, in severe cases, further surgical intervention [5].

Accurate and reliable documentation of prospective cohorts is a prerequisite for providing evidence regarding post-operative outcomes of ARCR including POSS. These data can be used for the development of clinical prediction models (CPM) allowing individual outcome predictions. Choice of the factors specifying CPM should rely on prior comprehensive systematic reviews and expert assessment [6]. The current literature reported limitations in the published evidence related to prognostic factors for structural or clinical outcomes of ARCR [2, 7–12]. We therefore set out to systematically review the literature to synthesize the evidence on prognostic factors for POSS after ARCR. Our objective was to map prognostic factors for the occurrence of POSS in patients after ARCR.

## Methods

This systematic review was written according to Preferred Reporting Items for Systematic Review and Meta-Analysis (PRISMA) reporting guidelines [13] and with the help of the Checklist for Critical Appraisal and data extraction for systematic reviews of prediction modeling studies for prognostic factors (CHARMS-PF) [14]. Risk of bias was assessed with the Quality in Prognosis Studies (QUIPS) tool [15] and the quality of evidence was graded with an adaptation of the GRADE framework applied to prognostic factor evidence [16]. The protocol was registered in PROSPERO on August 24, 2020 (registration number: CRD42020199257).

### Eligibility criteria

Longitudinal studies of patients with rotator cuff tear treated by primary ARCR were searched. We selected studies reporting on at least one prognostic factor for the occurrence of POSS, whatever definitions were used. Studies written in another language than English, French, or German, with a clinical follow-up of less than 6 months, on patients with irreparable tears, or revision operations were excluded.

### Information sources and search algorithm

The search strategies were developed by two information specialists (including CAH) and peer-reviewed by a third information specialist. Text word synonyms and database-specific subject headings for rotator cuff tear and arthroscopic repair surgery were used to search the electronic databases Embase (Elsevier), Medline (Ovid), and Scopus (Elsevier) without language restriction but excluding conference abstracts (Additional file 1; last search February 12, 2020). Since surgical rotator cuff repairs substantially evolved around 2013/2014 [17] and recent systematic reviews already summarized the evidence related to prognostic factors for ARCR patient outcomes [2, 7–12], the search results were limited to records published in 2014 and onwards. The final search string was written and optimized in embase.com syntax and translated for the other databases using a macro [18] and the systematic review accelerator [19], respectively. To complement the results of direct database searching, we screened the bibliographic references of all included articles as well as the citing articles of those that were indexed in Scopus or the Web of Science (November 23, 2020). The bibliographic references of identified systematic and narrative reviews on ARCR were also screened as an additional source. References were exported to Endnote X9 (Clarivate Analytics Philadelphia, PA USA) and deduplicated using the Bramer method [20].

### Study selection and data collection

The search results were screened independently by two reviewers (LM and TS) based on reference titles and abstracts. References that were not excluded by agreement were then retrieved in full text and assessed independently for eligibility (LM and TS).

Two review authors (either LM, TS, ML, or RL) independently extracted data from selected studies following an adapted version of the CHARMS-PF [14]. Any disagreements were resolved by consensus or involved arbitration by the last author (LA). Extraction items are listed in Additional file 2.

### Risk of bias assessment

Two review authors (either LM, TS, ML or RL) independently assessed the risk of bias of included studies after data extraction using the Quality in Prognosis Studies (QUIPS) tool [15]. Any disagreements were resolved by consensus or involved arbitration by the last author (LA). We agreed on a series of pre-defined key characteristics for the description of the population (tear pattern and tear etiology), the intervention (number of surgeons involved and repair technique), and the rehabilitation protocol (duration of post-operative immobilization) to guide our judgment when assessing the risk of bias for the Study participation item. The studies reporting only a part of univariable or bivariable effect estimates were all considered as having a high risk of bias regarding the statistical analysis and reporting item.

### Summary measures and synthesis of results

Effect estimates were reported as described in individual studies. Whenever possible, odds ratios (OR) and their confidence intervals were calculated (i.e. the number of events and non-events per variable and outcome group were reported). When needed, effect estimates were inverted by applying a simple inverse function to help us in interpreting the results of a given factor. A meta-analysis was performed if more than three studies assessed the association between POSS and the same prognostic factor estimate.

### Quality of evidence

As suggested by Riley et al. [21], we graded the quality of evidence related to prognostic factors using an adaptation of the GRADE framework [16]. This instrument contained six domains contributing to low quality including the phase of investigation (confirmatory or explanatory), study limitations, inconsistency across studies, indirectness (according to the review question), within (sample size, number of events per outcome) and across (number of studies and number of participants per study) study imprecision, and publication bias. Two additional domains were considered for higher quality of evidence: presence of moderate or large effect and exposure-gradient response.

### Prognostic factor terminology

When extracting data, a prognostic factor was understood as *“any variable that, among people with a given health condition (i.e. a start point), is associated with (the risk of) a subsequent clinical outcome (i.e. an endpoint). Different values (or categories) of a prognostic factor are associated with a better or worse prognosis.”* [21]

In the present review, we defined a factor as probably prognostic when, overall, authors of individual studies reported the same direction of association with at least a low quality of evidence (as ranked with the GRADE framework [16]).

## Results

### Study selection

**Fig. 1:**
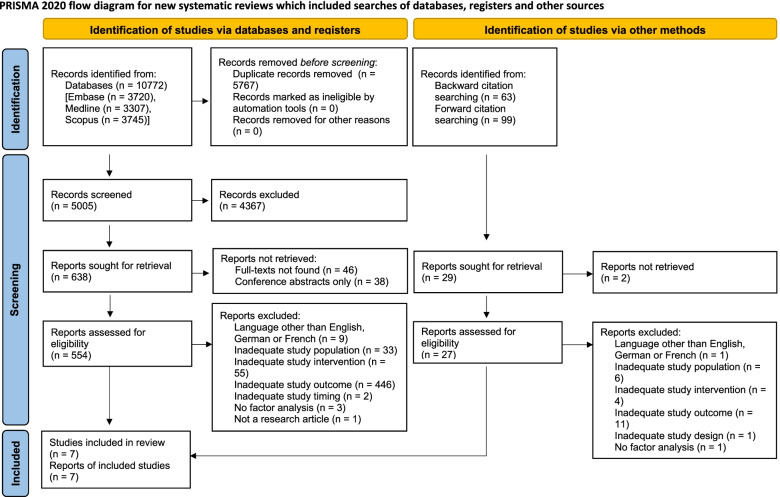
Selection of included studies (PRISMA 2020 Flow Diagram)

From 5005 initial records screened on titles and abstracts, 554 full-text articles were assessed for eligibility. After full-text screening, five studies were eligible for inclusion [22–26]. Backward and forward citation tracking on these as well as on seven [27–33] topical reviews that were flagged during title/abstract screening identified 162 additional potentially eligible records, two of which were included in the review [34, 35]. Most of the excluded full-text articles did not examine an adequate study outcome (*N* = 446). Study selection is summarized in Fig. 1.

### Study characteristics

All the studies were published between 2016 and 2020 and involved 23 257 patients across five countries (United States of America [23, 24], South Korea [25, 26], Australia [22], Japan [35], and Italy [34]).

#### Participants

The authors of three studies included patients with isolated supraspinatus rotator cuff tears [22, 26, 34]. In the other four studies, the type of tears was not reported [23–25, 35] (Table 1). Whereas one study included both degenerative and traumatic tears [22], another study included only degenerative tears [34]. In the other studies, the tear etiology was not precisely described [23–26, 35]. Patients with concomitant shoulder pathologies such as acromioclavicular arthritis, biceps pathologies, or shoulder instability requiring treatments were excluded in four studies [24–26, 34].

**Table 1:** Characteristics of included studies

Author	Year	Country	Design	Number of patients	Population	Intervention	Rehabilitation protocol
Cho, C.H.	2015	South Korea	P	80	Patients with types of tear patterns and etiology that were not precisely described; Exclusion of patients with workers compensation claims, or requiring additional procedures (AC arthritis, biceps pathologies)	One surgeon involved, repair technique not precisely described	Post-operative immobilization not precisely described. Active range of motion started at 6 weeks after surgery. Strengthening exercises started after 3 months, and sport activities from 6 months
Cho, N.S.	2015	South Korea	R	335	Patients with full-thickness supraspinatus tears, with fatty infiltration < 2, etiology not precisely described; Exclusion of patients with workers compensation claims, or requiring additional procedures (AC arthritis, biceps pathologies)	One surgeon involved, suture-bridge technique used	Post-operative immobilization not precisely described. Active range of motion started at 6 weeks after surgery. Strengthening exercises started after 6 weeks, and sport activities from 6 months
Tan, M.	2016	Australia	R	1300	Patients with supraspinatus tears, and both degenerative or traumatic tears; No specific exclusion criteria	One surgeon involved, single-row repair technique used	Post-operative immobilization in a sling with a small abduction pillow. Active range of motion started at 6 weeks after surgery. Strengthening exercises started after 3 months, and sport activities from 6 months
Burrus, M.T	2019	U.S.A.	R	19229	Patients with types of tear patterns and etiology that were not precisely described; Exclusion of patients requiring additional procedures (AC arthritis, biceps pathologies, and instability)	Number of surgeons involved and repair technique not precisely described	Rehabilitation procedure not precisely described
Harada, G.K.	2019	U.S.A.	R	1881	Patients with types of tear patterns and etiology that were not precisely described; No specific exclusion criteria	Number of surgeons involved and repair technique not precisely described	Rehabilitation procedure not precisely described
Cucchi, D.	2020	Italy	P	237	Patients with degenerative supraspinatus tears; Exclusion of patients requiring additional procedures (AC arthritis, biceps pathologies, and instability)	One surgeon involved, single-row repair technique used	Post-operative immobilization in a sling. Passive range of motion started after 1 month. Active range of motion started after reaching full passive range of motion. Strengthening exercises started after 3 months. Start of usual sport activities not precisely described
Takahashi, R.	2020	Japan	R	195	Patients with types of tear patterns and etiology that were not precisely described; Exclusion of patients requiring additional procedures (instability)	One surgeon involved, suture-bridged repair technique used	Post-operative immobilization in an abduction brace for 4 weeks. Passive range of motion started directly after the operation. Active range of motion started after 4 weeks. Strengthening exercises started after 6 weeks. Start of usual sport activities after 6 months

*AC* Acromioclavicular, *P* prospective, *R* retrospective, *U.S.A.* United States of America

#### Intervention

In five studies, authors reported outcome results for a single surgeon [22, 25, 26, 34, 35]. In the two remaining studies, the number of surgeons involved was not stated [23, 24] (Table 2). Either single-row [22, 34] or suture-bridge [25, 35] repair techniques were used. In the three remaining studies, the repair technique was not reported [23, 24, 26].

**Table 2:** Outcomes and statistical analyses of included studies

Author	Year	POSS definition	Time point (months)	Type	Value^**α**^	Statistical analysis methods
Tan, M.	2016	Part of a scale (L'Insalata questionnaire) describing perceived shoulder stiffness [36]	6	Categorized^μ^	-	Bivariable analysis using a 2-way between subject analysis of variance without considering cofounding
Cucchi, D.	2020	Part of range of motion parameters. Defined as forward flexion < 100°, or external rotation with arm at side < 10°, or external rotation with arm in 90° of abduction < 30°	3 - 6	Dichotomous	19/237 (8.01%)	Multivariable analysis using a logistic regression model specified with factors included if their influence was significant in the preliminary univariable analysis
Takahashi, R.	2020	Part of range of motion parameters. Defined as forward flexion < or equal 100°, and external rotation with arm at side < or equal to 10° and internal rotation < or equal to L5	6	Dichotomous	1/195 (0.51%)	Univariable analysis using Fisher's exact test without considering confounding
Burrus, M.T	2019	Requiring manipulation under anesthesia or a lysis of adhesion	9	Dichotomous	232/19229 (1.21%)	Multivariable database analysis using a logistic regression model specified with risk factors identified in the literature and controlling for several other cofounders
Cho, C.H.	2015	Part of range of motion parameters. Defined as forward flexion of < 120°, and external rotation with arm at side < 30°	12	Dichotomous	7/80 (8.75%)	Univariable analysis using a chi-square test without considering confounding
Harada, G.K.	2019	Requiring manipulation under anesthesia, such as capsular contracture release, shoulder joint, or arthroscopy	12	Dichotomous	73/1881 (3.88%)	Multivariable database analysis using a logistic regression model specified with factors chosen by the authors
Cho, N.S.	2015	Part of range of motion parameters. Defined as forward flexion < 120° and external rotation with arm at side < 30° and internal rotation < L3	24	Dichotomous	21/335 (6.27%)	Univariable analysis using a chi-square test without considering confounding

*L3* Third lumbar vertebra, *L5* Fifth lumbar vertebra, *POSS* Post-operative shoulder stiffness

α: for dichotomized outcomes, the value for the outcome was the prevalence at the given timepoint (i.e. the number of events over the number of patients analyzed in the study), μ: categories for L’Insalata questionnaire [36]: 0 = none, 1 = little, 2 = moderately, 3 = quite, 4 = very

#### Study design and outcome

Two studies were prospectively conducted [26, 34]. Four studies defined POSS based on range of motion parameters yet with different thresholds used [25, 26, 34, 35]. Resulting from the analysis of large registry databases, two studies defined POSS as an event requiring manipulation under anesthesia. However, the indication for such manipulation was not defined [23, 24]. One study used the responses to a single question of the L’Insalata questionnaire [36] describing the perceived POSS [22]. POSS event rate ranged between 0.51% [35] and 8.75 % [26] within a time period ranging from 6 months [22, 34, 35] to 24 months [25].

#### Statistical analysis methods

Three studies conducted a multivariable analysis using logistic regression models, specified with factors identified in the literature [24], chosen by the authors themselves [23], or with factors significantly associated with the outcome in the univariable analysis [34].

The four other studies reported a univariable or a bivariable analysis without considering potential confounding, using standard tests to compare groups (Fisher’s exact test or Chi-square test) [25, 26, 35] or a subject analysis of variance [22].

### Prognostic factor findings – results of individual studies

Overall, 22 potential prognostic factors were identified (see Table 3). Socio-demographic factors (such as age or sex), co-morbidities (like body mass index (BMI), smoking status, diabetes, or hypothyroidism), or injury characteristics (traumatic onset), investigated in at least two different studies, are presented below in separate sections (see Additional file 3). Other factors that were reported in only one study, respectively, are presented in the section “other factor”.

**Table 3:** Summary of prognostic factors findings for the occurrence of post-operative shoulder stiffness

Factor category	Probably prognostic	Requiring further analyses
**Patient-related**	Age [24, 34], Sex [24, 26, 34]	BMI [24, 34], Chronic pulmonary disease [34], Depression or anxiety [34], Diabetes [24, 25, 34, 35], Dyslipidemia [34], Gastroesophageal reflux disease [34], Hyper/hypo-thoiroidism [34], Hypercholesterolemia [34], Hypertension [34], Relatives with diabetes [34], Relatives with shoulder stiffness [34], Smoking status [24, 34], Vitamin D deficiency [23]
**Disease-related**		Dominance affected side [34], Preoperative shoulder stiffness [34], Systematic lupus erythematosus [24], Tear size [34], Traumatic onset [22]
**Procedure-related**		Symptom duration [22]

#### Age

Increased age emerged as a protective factor against POSS, with an association reported in two independent multivariable analyses [24, 34]. Yet, authors used different ways to handle this factor, either dichotomized (OR = 0.5 [0.4 ; 0.6] for POSS occurrence in the group of patients over 50 years old) [24] or kept continuous (OR = 0.9 [0.8 ; 0.9] for POSS occurrence with age increases by one year unit) [34]).

#### Body Mass Index (BMI)

None of the two studies assessing BMI as a factor did report a significant association with POSS (OR = 0.7 [0.37 ; 1.41] for underweight vs. no underweight and OR = 1.12 [0.9 ; 1.4] for overweight vs. no overweight [24], or p = 0.114 in univariable analysis [34]). There is currently no evidence supporting an association of BMI with the occurrence of POSS.

#### Diabetes

Reported results regarding the association between diabetes and the occurrence of POSS were inconsistent and the prognostic value of diabetes was unclear. One study reported that type I diabetes was significantly associated with POSS (OR = 2.7 [2.0 ; 3.7]) [24], whereas type II diabetes was not (OR = 0.9 [0.7 ; 1.1]) [24]. None of the three studies with univariable analyses reported a significant univariable testing (p > 0.254) [25, 34, 35].

#### Male sex

Male sex emerged as a probable protective factor against the occurrence of POSS, as its reported associations were consistent in two multivariable analyses [24, 34] (OR = 0.5 [0.4 ; 0.6] and OR = 0.1 [0.0 ; 0.6] for male sex) [24, 34]. An independant univariable analysis, however, did not reach statistical significance (*p* = 0.205) [26].

#### Hypothyroidism

The prognostic value of hypothyroidism remained unclear, as only one study reported that hypothyroidism was significantly associated with a higher risk of POSS [24] (OR = 1.3 [1.1 ; 1.6]), whereas the other univariable analysis did not reach statistical significance (*p* > 0.5) [34].

#### Smoking

The prognostic value of smoking status remained unclear, as the results of a multivariable analysis indicated that smoking was significantly associated with a lower risk of POSS (OR = 0.5 [0.36 ; 0.63]) [24] and in the other study, the univariable analysis was not statistically significant (*p* = 0.091) [34].

#### Other factors

Concomitant comorbidities (such as gastroesophageal reflux disease, systematic lupus erythematosus, vitamin D deficiency) were found to be prognostic factors associated with a higher risk of occurrence of POSS, yet with different statistical analyses [23, 24, 34]. A traumatic onset compared to degenerative tears was found to be associated with an increased risk of POSS [22]. The association between symptom duration and the risk of POSS was statistically significant, but the direction of this association was not reported [22].

### Quality of evidence

The quality of the evidence of our results was low (for increased age, male sex) or very low (for the other 20 prognostic factors), mostly due to the small number of included studies and inconsistencies across reported prognostic factor estimates (diabetes, smoking, hypothyroidism, etc.) (see Additional file 4).

### Risk of bias within studies

All included studies suffered from a high overall risk of bias, resulting from being judged at a high risk of bias in at least one of six bias domains (Table 4). Regarding individual bias domains, all included studies suffered from a moderate or a high risk of bias regarding study participation. A lack of clear description of the investigated prognostic factors impacted two studies [24, 34]. Only two studies addressed potential confounding by including factors in their final multivariable models identified in the literature [23, 24]. Only one study reported both univariable and multivariable effect estimates for all the prognostic factors examined [24].

**Table 4:** Risk of bias of included studies

Author	Year	Study participation	Study attrition	Prognostic factor measurement	Outcome measurement	Study confounding	Statistical analysis and reporting	Overall risk of bias
Cho, C.H.	2015	High	Low	Low	Low	High	High	High
Cho, N.S.	2015	Moderate	High	Low	Moderate	High	High	High
Tan, M.	2016	Moderate	Moderate	Moderate	High	High	High	High
Burrus, M.T	2019	Moderate	Moderate	High	Low	Low	Moderate	High
Harada, G.K.	2019	High	High	Moderate	Low	Low	High	High
Cucchi, D.	2020	Moderate	Low	High	High	High	High	High
Takahashi, R.	2020	High	High	Moderate	Moderate	High	High	High

### Analyses of the set of factors specifying multivariable models

Three studies presented a set of prognostic factors to be considered in multivariable models (Table 5) [23, 24, 34]. Age and sex were found in all these sets. However, the authors included different comorbidities in their final models [23, 24, 34]. The selection process was different in the three studies. One study was fully data-driven [34], whereas the two other studies pre-specified their models [23, 24], with one study using the existing literature [24].

**Table 5:** Set of factors of multivariable models

Author	Year	Set of factors	Selection process
Cucchi, D.	2020	Age, sex, presence of gastroesophageal reflux disease, and depression and anxiety	Factors significant in univariable analysis
Burrus, M.T.	2019	Age, sex, body mass index, tobacco use, diabetes, thyroid disorders and systematic lupus erythematosus. Control of several comorbidities (alcohol use, depression, hyperlipidemia, hypertension, peripheral vascular disease, coronary artery disease, congestive heart failure, chronic kidney disease, current hemodialysis use, and chronic lung disease)	Pre-specification of the model, using existing literature
Harada, G.K.	2019	Age, sex, Charlson Comorbity Index and vitamin D levels	Not precisely described

### Meta-analysis and risk of bias across studies

Considering the small number of included studies in the present review, we could not perform meta-analysis, evaluate the risk of bias across studies, or conduct any additional subgroup analysis.

## Discussion

Twenty-two potential prognostic factors for POSS after ARCR were identified in the present review. The best available evidence pointed to increased age and male sex as probable prognostic factors decreasing the risk of occurrence of POSS. Associations of various comorbidities such as diabetes, hypothyroidism, and BMI and smoking status with POSS were also reported but do require further analyses to determine their prognostic value.

Our findings with regard to the low methodological quality of included studies were consistent with previous systematic reviews [7, 12]. Of note, older age (more than 50 years old) was already found to be a protective factor for the occurrence of POSS [31]. Nevertheless, this association is still puzzling. On the one hand, older patients tend to have larger tears, for which repairs are thought to be prone to increased initial joint tightness [37], possibly also due to reduced initial tendon length [38]. Repair of larger and more retracted tears may also require advanced surgical dissection that is believed to trigger postoperative fibrosis. On the other hand, repairs of smaller and partial rotator cuff tears –which occur more frequently in younger patients, have been shown to be associated with a higher rate of POSS in previous studies [39]. The high incidence of preoperative rotator interval fibrosis observed in partial tears may partly explain this association [40]. The protective effect of male sex has also been previously identified in investigations on primary adhesive capsulitis [41–43]. Testosterone may inhibit the transforming growth factor beta signaling pathway, which mediates capsular contractions and adhesions seen in POSS [44].

Knowledge of prognostic factors for POSS may help clinicians to tailor patient-specific rehabilitation schemes, e.g. female and younger patients may benefit from limited immobilization [45] and more rapid rehabilitation strategies [46] in the postoperative period. Liberal use of postoperative steroid injections may also be considered in these patients [47].

### Limitations

First, our selection criteria based on study language (studies published in French, English or German) might have affected our review results, as we could not assess the content of 10 specific records, which could have changed our findings. Second, the well-known lack of a universal definition of POSS [33] limited the interpretation of our results, as various outcome definitions were used (event requiring manipulation under anesthesia, range of motion parameters, and perceived shoulder stiffness) measured at different endpoints (ranging from 6 months up to 24 months). Third, regarding the statistical analysis, interpretation of our results suffered from the lack of proper multivariable analyses and reports, usually ensuring that a factor has a relevant prognostic value while considering a pre-specified set of factors already known as confounders [6].

## Conclusions

Male sex and increased age emerged from the present review as probable prognostic factors decreasing the risk of POSS after ARCR. The high risk of bias of included studies, however, dramatically lowered the strength of evidence. The factors identified as probably prognostic in the present review do require further analyses to draw stronger conclusions regarding their prognostic value in different settings. This requires the implementation of registries or prospective cohort studies following high methodological standards in the field of arthroscopic rotator cuff repair. To establish a reliable pre-specified set of factors for CPM predicting POSS, our review results do need to be complemented with an expert’s opinion.

### Three take-home messages:

1. Male sex and increased age are probable prognostic factors decreasing the risk of POSS. BMI, smoking status, and hypothyroidism require further analyses to be confirmed as prognostic factors.
2. Low methodological quality of included studies impaired us from drawing clear conclusions.
3. Further clinical prediction model development and prognostic factor analyses regarding post-operative shoulder stiffness and relying on prospective and well-designed cohort studies should be conducted.

## Supplementary Information

**Additional file 1.** Search strategies

**Additional file 2.** Extracted data items

**Additional file 3.** Prognostic factors estimates of included studies

**Additional file 4.** GRADE framework applied to prognostic factors findings

## Data Availability

The electronic database used to extract the data from individual studies and the code associated with the design of the tables will be uploaded on a GitHub repository once the systematic review for the other outcomes as described on PROSPERO will be completed [48].

## Abbreviations

ARCR: Arthroscopic Rotator Cuff Repair
CHARMS-PF: Checklist for Critical Appraisal and data extraction for systematic reviews of prediction modeling studies for prognostic factors
CPM: Clinical Prediction Model
DMT: Diabetes Mellitus Type
PRISMA: Preferred Reporting Items for Systematic Review and Meta-Analysis
POSS: Post-operative Shoulder Stiffness
QUIPS: Quality in prognosis study
US: Ultrasound
MRI: Magnetic Resonance Imaging
