# Supplementary material for: Prognostic factors for the occurrence of post-operative shoulder stiffness after arthroscopic rotator cuff repair: a systematic review
Source: BMC Musculoskelet Disord. 2022 Jan 28;23:99. doi: 10.1186/s12891-022-05030-4 (PMC8800355; doi:10.1186/s12891-022-05030-4)
Supplement: Supplementary file 1 — Additional file 1. Search strategies [file 12891_2022_5030_MOESM1_ESM.docx]

**Additional file 1:** search strategies

Embase.com

('rotator cuff injury'/de OR 'rotator cuff rupture'/de OR 'rotator cuff repair'/de OR 'arthroscopic rotator cuff repair'/de OR 'supraspinatus tear'/de OR 'supraspinatus tendon tear'/de OR 'supraspinatus tendon rupture'/de OR 'supraspinatus tendinopathy'/de OR 'subscapularis tear'/de OR 'subscapularis tendon tear'/de OR 'subscapularis tendon rupture'/de OR (((cuff OR subscapularis OR supraspinatus OR infraspinatus OR 'teres minor') NEAR/4 (disease OR syndrome OR disorder* OR injur* OR lesion* OR tear* OR torn OR rupture* OR patholog* OR tendinopath* OR degeneration)) OR (('rotator cuff' OR subscapularis OR supraspinatus OR infraspinatus OR 'teres minor') NEAR/3 (surgery OR surgical OR repair* OR operation* OR operative OR reconstruct*))):ab,ti OR (('rotator cuff'/exp OR 'subscapularis muscle'/de OR 'subscapularis tendon'/de OR 'supraspinatus muscle'/de OR 'supraspinatus muscle tendon'/de OR 'supraspinatus tendon'/de OR 'infraspinatus muscle'/de OR 'infraspinatus tendon'/de) AND (disease OR syndrome OR disorder* OR injur* OR lesion* OR tear* OR torn OR rupture* OR patholog* OR tendinopath* OR degeneration OR surgery OR surgical OR repair* OR operation* OR operative OR reconstruct*):ab,ti))

AND

('surgery'/de OR 'arthroscopic surgery'/de OR 'tendon surgery'/de OR 'shoulder surgery'/de OR 'rotator cuff repair'/de OR 'arthroscopic rotator cuff repair'/de OR (surgery OR surgical OR repair* OR operation* OR operative OR reconstruct*):ab,ti)

AND

(2014:py OR 2015:py OR 2016:py OR 2017:py OR 2018:py OR 2019:py OR 2020:py)

NOT

(('animal'/de OR 'animal experiment'/exp OR 'nonhuman'/de) NOT ('human'/exp OR 'human experiment'/de))

NOT

[conference abstract]/lim

Medline (Ovid)

(rotator cuff injuries/ OR (((cuff OR subscapularis OR supraspinatus OR infraspinatus OR teres minor) ADJ4 (disease OR syndrome OR disorder* OR injur* OR lesion* OR tear* OR torn OR rupture* OR patholog* OR tendinopath* OR degeneration)) OR ((rotator cuff OR subscapularis OR supraspinatus OR infraspinatus OR teres minor) ADJ3 (surgery OR surgical OR repair* OR operation* OR operative OR reconstruct*))).ab,ti. OR (rotator cuff/ AND (disease OR syndrome OR disorder* OR injur* OR lesion* OR tear* OR torn OR rupture* OR patholog* OR tendinopath* OR degeneration OR surgery OR surgical OR repair* OR operation* OR operative OR reconstruct*).ab,ti.))

AND

(surgical procedures, operative/ OR arthroscopy/ OR general surgery/ OR (surgery OR surgical OR repair* OR operation* OR operative OR reconstruct*).ab,ti.)

AND

("2014" or "2015" or "2016" or "2017" or "2018" or "2019" or "2020").yr.

NOT

(exp animals/ NOT humans/)

Scopus

TITLE-ABS ( ( ( ( cuff OR subscapularis OR supraspinatus OR infraspinatus OR "teres minor" ) W/4 ( disease OR syndrome OR disorder* OR injur* OR lesion* OR tear* OR torn OR rupture* OR patholog* OR tendinopath* OR degeneration ) ) OR ( ( "rotator cuff" OR subscapularis OR supraspinatus OR infraspinatus OR "teres minor" ) W/2 ( surgery OR surgical OR repair* OR operation* OR operative OR reconstruct* ) ) )

AND

( surgery OR surgical OR repair* OR operation* OR operative OR reconstruct* ) )

AND

( LIMIT-TO ( PUBYEAR , 2020 ) OR LIMIT-TO ( PUBYEAR , 2019 ) OR LIMIT-TO ( PUBYEAR , 2018 ) OR LIMIT-TO ( PUBYEAR , 2017 ) OR LIMIT-TO ( PUBYEAR , 2016 ) OR LIMIT-TO ( PUBYEAR , 2015 ) OR LIMIT-TO ( PUBYEAR , 2014 ) )
