# Supplementary material for: Prognostic factors for the occurrence of post-operative shoulder stiffness after arthroscopic rotator cuff repair: a systematic review
Source: BMC Musculoskelet Disord. 2022 Jan 28;23:99. doi: 10.1186/s12891-022-05030-4 (PMC8800355; doi:10.1186/s12891-022-05030-4)
Supplement: Supplementary file 2 — Additional file 2. Extracted data items [file 12891_2022_5030_MOESM2_ESM.docx]

**Additional file 2**: extracted data items

| Item N° | Specification |
| --- | --- |
| Study characteristics | |
| 1 | Title, first author and year of publication |
| 2 | Country of research group |
| 3 | Research design (prospective vs retrospective) |
| Participants | |
| 4 | Inclusion criteria |
| 5 | Exclusion criteria |
| 6 | Number of included patients |
| 7 | Number of studied shoulders |
| 8 | Patients’ average age |
| 9 | Patients’ sex ratio |
| Intervention | |
| 10 | Surgical intervention definition |
| 11 | Rehabilitation protocol |
| Follow-up | |
| 12 | Duration of follow-up (minimum-maximum) |
| Outcomes |  |
| 13 | Number of studied outcomes |
| Per outcome | |
| 14 | Targeted outcome type (repair integrity, shoulder function, shoulder stiffness) |
| 15 | Definition or scale used |
| 16 | Type (continuous, dichotomous, categorized, unclear) |
| 17 | Assessment time point (in months) |
| 18 | Blinding measurement (yes/no) |
| 19 | Analysis of risk factors (univariable vs multivariable) |
| Per prognostic factor | |
| 20 | Definition |
| 21a | Type (continuous, dichotomous, categorized, unclear) |
| 21b | Classes (in case of dichotomous or categorized) |
| 23 | Method (univariable, multivariable or both) |
| 24a | Effect estimate type (result of a test, odds ratio, risk ratio, mean difference, coefficients) |
| 24b | Effect estimate (e.g. odds ratio = 2.05) |
| 24c | Effect estimate accuracy (e.g. odds ratio confidence interval 95% = [2.01 ; 2.09]) |
| 24d | p-value (e.g. p = 0.05) |
