# Supplementary material for: Prognostic factors for the occurrence of post-operative shoulder stiffness after arthroscopic rotator cuff repair: a systematic review
Source: BMC Musculoskelet Disord. 2022 Jan 28;23:99. doi: 10.1186/s12891-022-05030-4 (PMC8800355; doi:10.1186/s12891-022-05030-4)
Supplement: Supplementary file 3 — Additional file 3. Prognostic factors estimates of included studies [file 12891_2022_5030_MOESM3_ESM.docx]

**Additional file 3**: prognostic factors estimates of included studies

|  |  |  |  |  | **Univariable** | | | | **Multivariable** | | | |
| --- | --- | --- | --- | --- | --- | --- | --- | --- | --- | --- | --- | --- |
| **Author** | **Year** | **Prognostic factor studied** | **Definition** | **Type** | **Reported** | **Effect estimate** | **95% CI** | **p value** | **Reported** | **Effect estimate** | **95% CI** | **p value** |
| Burrus, M.T | 2019 | Age | More than 50 years old | D | - | - | - | - | OR | 0.5 | [0.4 ; 0.6] | < 0.0001 |
| Cucchi, D. | 2020 | Age | - | C | Test | S | - | 0.0051 | OR | 0.9 | [0.8 ; 0.9] | 0.001 |
| Burrus, M.T | 2019 | BMI | Overweight (BMI > 30 kg/ m2) | D | - | - | - | - | OR | 1.12 | [0.9 ; 1.4] | 0.239 |
| Burrus, M.T | 2019 | BMI | Underweight (BMI < 19 kg/m2) | D | - | - | - | - | OR | 0.7 | [0.4 ; 1.4] | 0.335 |
| Cucchi, D. | 2020 | BMI | - | C | Test | NS | - | 0.114 | - | - | - | - |
| Cho, N.S. | 2015 | Diabetes | Usual diabetes definition | D | Test | NS | - | 0.254 | - | - | - | - |
| Takahashi, R. | 2020 | Diabetes | Usual diabetes definition | D | Test | NS | - | 0.344 | - | - | - | - |
| Burrus, M.T | 2019 | Diabetes | Usual diabetes mellitus type I definition | D | - | - | - | - | OR | 2.7 | [2.0 ; 3.6] | < 0.0001 |
| Burrus, M.T | 2019 | Diabetes | Usual diabetes mellitus type II definition | D | - | - | - | - | OR | 0.9 | [0.7 ; 1.0] | 0.193 |
| Cucchi, D. | 2020 | Diabetes | NA | D | OR | 0.5 | [0.0 ; 3.8] | 0.703 | - | - | - | - |
| Cho, C.H. | 2015 | Sex | Usual sex definition | D | Test | NS | - | 0.205 | - | - | - | - |
| Burrus, M.T | 2019 | Sex | Usual sex definition | D | - | - | - | - | OR | 0.5 | [0.4 ; 0.6] | < 0.0001 |
| Cucchi, D. | 2020 | Sex | Usual sex definition | D | Test | S | - | 0.0005 | OR | 0.1 | [0.0 ; 0.6] | 0.012 |
| Cucchi, D. | 2020 | Smoking | NA | D | OR | 0.4 | [0.1 ; 1.0] | 0.091 | - | - | - | - |
| Burrus, M.T | 2019 | Smoking | NA | D | - | - | - | - | OR | 0.5 | [0.4 ; 0.6] | < 0.0001 |
| Cucchi, D. | 2020 | Chronic obstructive pulmonary disease | NA | D | OR | 0.0 | [0 ; 0] | > 0.999 | - | - | - | - |
| Cucchi, D. | 2020 | Depression or anxiety | NA | D | OR | 3.74 | [1.2 ; 11.5] | 0.0305 | OR | 2.1 | [0.6 ; 7.7] | 0.272 |
| Cucchi, D. | 2020 | Dyslipidemia | NA | D | OR | 1.1 | [0.2 ; 4.8] | 0.999 | - | - | - | - |
| Cucchi, D. | 2020 | Gastroesophageal reflux disease | Diagnosis established using GerdQ questionnaire | D | OR | 4.71 | [1.8 ; 12.4] | 0.0305 | OR | 5.3 | [1.7 ; 16.7] | 0.005 |
| Cucchi, D. | 2020 | Hypercholosterolemia | NA | D | OR | 1.1 | [0.4 ; 3.3] | 0.784 | - | - | - | - |
| Cucchi, D. | 2020 | Hypertension | NA | D | OR | 0.4 | [0.1 ; 1.3] | 0.146 | - | - | - | - |
| Burrus, M.T | 2019 | Systematic lupus erythematosus | Systematic lupus erythematosus, not clearly stated | D | - | - | - | - | OR | 2.1 | [1.2 ; 3.5] | 0.004 |
| Harada, G.K. | 2019 | Vitamin D | Groups of vitamin D sufficiency defined with preoperative plasma 25D levels | D | - | - | - | - | OR | 1.2 | [1.0 ; 2.0] | 0.035 |
| Burrus, M.T | 2019 | Hypothyroidism | NA | D | - | - | - | - | OR | 1.3 | [1.0 ; 1.6] | 0.012 |
| Cucchi, D. | 2020 | Hyperthyroidism | NA | D | OR | 12.1 | [0.7 ; 200] | 0.154 | - | - | - | - |
| Cucchi, D. | 2020 | Hypothyroidism | NA | D | OR | 1.0 | [0.3 ; 3.5] | 0.724 | - | - | - | - |
| Cucchi, D. | 2020 | Relatives with diabetes | NA | D | OR | 0.7 | [0.2 ; 2.0] | 0.618 | - | - | - | - |
| Cucchi, D. | 2020 | Relatives with shoulder stiffness | NA | D | OR | 2.2 | [0.6 ; 8.4] | 0.207 | - | - | - | - |
| Cucchi, D. | 2020 | Affected side dominant | Usual dominance of affected side definition | D | OR | 0.4 | [0.1 ; 1.0] | 0.084 | - | - | - | - |
| Cucchi, D. | 2020 | Tear size | Not clearly stated, lesion dimension superior or inferior to 1 cm | Unclear | Test | NS | - | 0.999 | - | - | - | - |
| Cucchi, D. | 2020 | Preoperative shoulder stiffness | NA | D | Test | 1.5 | [0.2 ; 12.3] | 0.535 | - | - | - | - |
| Tan, M. | 2016 | Symptom duration (timing) | Duration of symptoms in months | C | Test | S | - | < 0.0001 | - | - | - | - |
| Tan, M. | 2016 | Traumatic onset | Traumatic patients defined as recalling a specific injury | D | Test | S | - | 0.008 | - | - | - | - |
| **Footnote**: 0 = no influence, BMI = Body mass index, C = Continuous, Cat = Categorized, D = Dichotomous, NA = Not clearly stated, NS = Not significant, OR = Odds ratio, S = Significant,  SLE = Systematic lupus erythematosus | | | | | | | | | | | | |
