# Supplementary material for: Prognostic factors for the occurrence of post-operative shoulder stiffness after arthroscopic rotator cuff repair: a systematic review
Source: BMC Musculoskelet Disord. 2022 Jan 28;23:99. doi: 10.1186/s12891-022-05030-4 (PMC8800355; doi:10.1186/s12891-022-05030-4)
Supplement: Supplementary file 4 — Additional file 4. GRADE framework applied to prognostic factors findings [file 12891_2022_5030_MOESM4_ESM.docx]

**Additional file 4**: GRADE framework applied to prognostic factors findings

|  |  |  | Univariate | | | Multivariate | | |  | GRADE factors | | | | | | |  |
| --- | --- | --- | --- | --- | --- | --- | --- | --- | --- | --- | --- | --- | --- | --- | --- | --- | --- |
| **Potential prognostic factors identified** | **Number of participants** | **Number of studies** | **+** | **0** | **-** | **+** | **0** | **-** | **Phase** | **Study limitations** | **Inconsistency** | **Indirectness** | **Imprecision** | **Publication bias** | **Moderate/large effet size** | **Dose effect** | **Overall quality** |
| Affected side dominant | 237 | 1 |  | 1 |  |  |  |  | 1 | ✕ | ✕ | ✓ | ✕ | ✕ | ✕ | ✕ | + |
| Increasing age | 19466 | 2 | 1 |  |  | 2 |  |  | 1 | ✕ | ✓ | ✕ | ✓ | ✕ | ✓ | ✕ | ++ |
| BMI | 19466 | 2 |  | 1 |  |  | 1 |  | 1 | ✕ | ✕ | ✕ | ✕ | ✕ | ✕ | ✕ | + |
| Chronic obstructive pulmonary disease | 237 | 1 |  | 1 |  |  |  |  | 1 | ✕ | ✕ | ✓ | ✕ | ✕ | ✕ | ✕ | + |
| Depression or anxiety | 237 | 1 |  |  | 1 |  | 1 |  | 1 | ✕ | ✕ | ✓ | ✕ | ✕ | ✕ | ✕ | + |
| Diabetes | 19996 | 4 |  | 3 |  |  | 1 | 1 | 1 | ✕ | ✕ | ✕ | ✕ | ✕ | ✕ | ✕ | + |
| Dyslipidemia | 237 | 1 |  | 1 |  |  |  |  | 1 | ✕ | ✕ | ✓ | ✕ | ✕ | ✕ | ✕ | + |
| Presence of gastroesophageal reflux disease | 237 | 1 |  |  | 1 |  |  | 1 | 1 | ✕ | ✕ | ✓ | ✕ | ✕ | ✓ | ✕ | + |
| Male sex | 19546 | 3 | 1 | 1 |  | 2 |  |  | 1 | ✕ | ✓ | ✕ | ✓ | ✕ | ✓ | ✕ | ++ |
| Hyperthyroidism | 237 | 1 |  | 1 |  |  |  |  | 1 | ✕ | ✕ | ✓ | ✕ | ✕ | ✕ | ✕ | + |
| Hypothyroidism | 19466 | 2 |  | 1 |  |  |  | 1 | 1 | ✕ | ✕ | ✕ | ✕ | ✕ | ✕ | ✕ | + |
| Hypercholosterolemia | 237 | 1 |  | 1 |  |  |  |  | 1 | ✕ | ✕ | ✓ | ✕ | ✕ | ✕ | ✕ | + |
| Hypertension | 237 | 1 |  | 1 |  |  |  |  | 1 | ✕ | ✕ | ✓ | ✕ | ✕ | ✕ | ✕ | + |
| Preoperative shoulder stiffness | 237 | 1 |  | 1 |  |  |  |  | 1 | ✕ | ✕ | ✓ | ✕ | ✕ | ✕ | ✕ | + |
| Relatives with diabetes | 237 | 1 |  | 1 |  |  |  |  | 1 | ✕ | ✕ | ✓ | ✕ | ✕ | ✕ | ✕ | + |
| Relatives with shoulder stiffness | 237 | 1 |  | 1 |  |  |  |  | 1 | ✕ | ✕ | ✓ | ✕ | ✕ | ✕ | ✕ | + |
| Smoking | 19466 | 2 | 1 |  |  | 1 |  |  | 1 | ✕ | ✓ | ✕ | ✓ | ✕ | ✓ | ✕ | + |
| Symptom duration (timing) | 1300 | 1 | Unclear | | | | | | 1 | Not assessable | | | | | | | |
| Systematic lupus erythematosus | 19229 | 1 |  |  |  |  |  | 1 | 1 | ✕ | ✕ | ✕ | ✕ | ✕ | ✓ | ✕ | + |
| Tear size | 237 | 1 |  | 1 |  |  |  |  | 1 | ✕ | ✕ | ✓ | ✕ | ✕ | ✕ | ✕ | + |
| Traumatic onset | 1300 | 1 |  |  | 1 |  |  |  | 1 | ✕ | ✕ | ✕ | ✕ | ✕ | ✕ | ✕ | + |
| Vitamin D deficiency | 1881 | 1 |  |  |  |  |  | 1 | 1 | ✕ | ✕ | ✕ | ✕ | ✕ | ✓ | ✕ | + |
| **Footnote**: For uni- and multivariate analyses: +, number of significant effects with a positive value (meaning the factor decreases the risk of occurrence of POSS); 0, number of non-significant effects; -, number of significant effects with a negative value (meaning the factor increases the risk of occurrence of POSS). For GRADE factors: ✓, no serious limitations; ✕, serious limitations (or not present for moderate/large effect size, dose effect); unclear, unable to rate item based on available information. For overall quality of evidence: +, very low; ++, low; +++, moderate; ++++, high. | | | | | | | | | | | | | | | | | |
